# A Guinea Pig Model of Airway Smooth Muscle Hyperreactivity Induced by Chronic Allergic Lung Inflammation: Contribution of Epithelium and Oxidative Stress

**DOI:** 10.3389/fphar.2018.01547

**Published:** 2019-01-24

**Authors:** Luiz Henrique César Vasconcelos, Maria da Conceição Correia Silva, Alana Cristina Costa, Giuliana Amanda de Oliveira, Iara Leão Luna de Souza, Fernando Ramos Queiroga, Layanne Cabral da Cunha Araujo, Glêbia Alexa Cardoso, Renato Fraga Righetti, Alexandre Sérgio Silva, Patrícia Mirella da Silva, Carla Roberta de Oliveira Carvalho, Giciane Carvalho Vieira, Iolanda de Fátima Lopes Calvo Tibério, Fabiana de Andrade Cavalcante, Bagnólia Araújo da Silva

**Affiliations:** ^1^Programa de Pós graduação em Produtos Naturais e Sintéticos Bioativos, Centro de Ciências da Saúde, Universidade Federal da Paraíba, João Pessoa, Brazil; ^2^Graduação em Farmácia, Departamento de Ciências Farmacêuticas, Centro de Ciências da Saúde, Universidade Federal da Paraíba, João Pessoa, Brazil; ^3^Programa de Pós graduação em Ciências (Fisiologia Humana), Instituto de Ciências Biológicas, Universidade de São Paulo, São Paulo, Brazil; ^4^Programa Associado de Pós graduação em Educação Física, Universidade Federal da Paraíba/Universidade do Pernambuco, João Pessoa, Brazil; ^5^Faculdade de Medicina FMUSP, Universidade de São Paulo, São Paulo, Brazil; ^6^Hospital Sírio Libanês, São Paulo, Brazil; ^7^Departamento de Educação Física, Centro de Ciências da Saúde, Universidade Federal da Paraíba, João Pessoa, Brazil; ^8^Departamento de Biologia Molecular, Centro de Ciências da Saúde, Universidade Federal da Paraíba, João Pessoa, Brazil; ^9^Departamento de Biofísica e Fisiologia, Instituto de Ciências Biológicas, Universidade de São Paulo, São Paulo, Brazil; ^10^Departamento de Morfologia/Centro de Ciências da Saúde/Universidade Federal da Paraíba, João Pessoa, Brazil; ^11^Departamento de Fisiologia e Patologia/Centro de Ciências da Saúde/Universidade Federal da Paraíba, João Pessoa, Brazil; ^12^Departamento de Ciências Farmacêuticas/Centro de Ciências da Saúde/Universidade Federal da Paraíba, João Pessoa, Brazil

**Keywords:** airways, asthma, contractile reactivity, relaxation, oxidative stress

## Abstract

Asthma is a heterogeneous disease of the airways characterized by chronic inflammation associated with bronchial and smooth muscle hyperresponsiveness. Currently, different murine models for the study of asthma show poor bronchial hyperresponsiveness due to a scarcity of smooth muscle and large airways, resulting in a failure to reproduce smooth muscle hyperreactivity. Thus, we aimed to standardize a guinea pig model of chronic allergic lung inflammation mimicking airway smooth muscle hyperreactivity observed in asthmatics (Asth). Animals were randomly divided into a control group (Ctrl), which received saline (0.9% NaCl), and the Asth group, subjected to *in vivo* sensitization with ovalbumin (OVA) nebulization. Morphological analysis was performed by hematoxylin-eosin staining. Bronchial hyperresponsiveness was evaluated by nebulization time in the fifth, sixth, and seventh inhalations (NT5-7) and tracheal isometric contractions were assessed by force transducer. Total antioxidant capacity was measured by the 2,2-diphenyl-1-picrylhydrazyl (DPPH) method and protein expression by Western blot. Histologically, the Asth group developed peribronchial cellular infiltrate, epithelial hyperplasia and smooth muscle thickening. After the fourth nebulization, the Asth group developed bronchial hyperreactivity. The trachea from the Asth group contracted after *in vitro* stimulation with OVA, differing from the Ctrl group, which showed no response. Additionally, airway smooth muscle hyperreactivity to carbachol and histamine was observed in the Asth group only in intact epithelium preparations, but not to KCl, and this effect was associated with an augmented production of reactive oxygen species. Moreover, lung inflammation impaired the relaxant potency of isoproterenol only in intact epithelium preparations, without interfering with nifedipine, and it was found to be produced by transforming growth factor-β negative modulation of β adrenergic receptors and, furthermore, big-conductance Ca^2+^-sensitive K^+^ channels. These effects were also associated with increased levels of phosphatidylinositol 3-kinases but not extracellular signal-regulated kinases 1/2 or phosphorylation, and augmented α-actin content as well, explaining the increased smooth muscle mass. Furthermore, pulmonary antioxidant capacity was impaired in the Asth group. Therefore, we developed a standardized and easy-to-use, reproducible guinea pig model of lung inflammation that mimics airway smooth muscle hypercontractility, facilitating the investigation of the mechanisms of bronchial hyperresponsiveness in asthma and new therapeutic alternatives.

## Introduction

Asthma is a chronic inflammatory disease of the airways in which many different innate and adaptive cells of the immune system act together with epithelial cells to promote excessive mucus production, airway wall remodeling, smooth muscle thickening and lumen narrowing (Lambrecht and Hammad, 2015), in addition to bronchial hyperresponsiveness, characterized as an increase in airway smooth muscle sensitivity and responsiveness to bronchoconstrictors (Baraldo et al., 2014) as well as a decrease in response to bronchodilator stimuli (Brehm et al., 2015).

Bronchial hyperresponsiveness results from some structural changes in airway morphology due to the inflammatory process (Amin, 2015). There is a strong relationship between Th2 cytokines and the pathognomonic airway changes characteristic of asthma remodeling. Among these changes, the most prominent feature is the increase in smooth muscle mass (Carroll et al., 1993) due to hypertrophy and hyperplasia, as well as the differentiation and migration of mesenchymal cells to the smooth muscle cell layer (Schmidt et al., 2003; Hirst et al., 2004; Bergeron et al., 2009). These phenomena are responsible for the increased contractility in asthma, the main causal factor of bronchial hyperresponsiveness (Lauzon and Martin, 2016).

Furthermore, early and late reactions in acute asthma involve changes in the control of airway smooth muscle function of neurogenic and non-neurogenic origin. Additionally, epithelial damage, thickening of the mucosa due to local vasodilation and edema and mucous secretions in the airway lumen are all involved in the development of airway hyperresponsiveness (Bousquet et al., 2004).

Asthma is also characterized by an overload of reactive oxygen species (ROS), which causes oxidative stress and changes in the functions of various components of the respiratory system (Morwood et al., 2005; Bishopp et al., 2017). The main ROS involved are superoxide anion (O_2_^-^), hydroxyl radicals (OH^-^) and hydrogen peroxide (H_2_O_2_), which lead to the formation of another variety of ROS (Loxham et al., 2014). In addition, asthma alters the mediators responsible for regulating airway smooth muscle tone, namely agonists, such as acetylcholine (ACh), histamine, prostaglandin D_2_ (PGD_2_), neurokinins and cysteinyl leukotrienes (cysLTs), which act by pharmacomechanical coupling in which binding to pharmacological receptors activates the phospholipase C effector system, leading to increased cytosolic Ca^2+^ levels and smooth muscle contraction (Pelaia et al., 2008; Alexander et al., 2013). This event can also be activated by electromechanical coupling, dependent on membrane depolarization and the opening of voltage-sensitive Ca^2+^ channels (Bolton, 1979).

Several asthma animal models have been established to investigate relevant pathological mechanisms. Mice have traditionally been used to elucidate the inflammatory mechanisms of respiratory diseases; however, mediators that regulate smooth muscle tone and determine bronchial responsiveness in these animals differ considerably from those found in humans (Canning, 2003). In addition, murine pulmonary structure is characterized by large and sparse airways in smooth muscle tissue (Zosky and Sly, 2007) as well as less responsiveness to several bronchoconstrictor stimuli implicated in the pathophysiology of asthma, such as histamine, cysLTs, neurokinins, and prostanoids, including PGD_2_ and thromboxane A_2_ (TxA_2_) (Canning, 2003). Differently, guinea pigs display greater proximity to humans in relation to smooth muscle response and autonomic reflex. They also release mainly histamine and cysLTs in response to mast cell stimulation, as well as showing a pharmacological receptor profile approaching humans,’ which explains the great similarity of responses after exposure to drugs (Ressmeyer et al., 2006).

Although there are many animal models of asthma, most of these models focus on acute inflammation based on allergen exposure for a short period of time (McMillan and Lloyd, 2004). In addition, many models fail to reproduce human airway hyperresponsiveness characteristics (Goldie et al., 1986).

Therefore, this study was conducted to develop a standardized guinea pig model of chronic allergic lung inflammation that mimics the characteristics of contractile hyperreactivity and relaxant hyporesponsiveness of airway smooth muscle, similar to that occurring in human asthma, thus providing a useful tool for the study of bronchial hyperresponsiveness mechanisms and the search for new antiasthmatic drugs.

## Materials and Methods

### Animals

Male and female adult guinea pigs (*Cavia porcellus*), weighing 300–500 g, were obtained from the Bioterium Professor Thomas George of Universidade Federal da Paraíba (UFPB). The animals were maintained on a 12-h light-dark cycle (lights on from 6 am to 6 pm), under controlled ventilation and temperature (21 ± 1°C) with free access to food (Presence^®^) and water. The experimental procedures were performed following the guidelines for the ethical use of animals in applied etiology studies (Sherwin et al., 2003), the Brazilian Guide for the Production, Maintenance or Use of Animals in Teaching or Scientific Research Activities, from Conselho Nacional de Controle de Experimentação Animal (CONCEA) (Silva et al., 2016), and approved by the Ethics Committee on Animal Use of UFPB (Protocol No. 0410/13).

### Chemicals

Calcium chloride (CaCl_2_), magnesium sulfate (MgSO_4_), potassium chloride (KCl) and sodium chloride (NaCl) were purchased from Vetec Química Fina Ltda. (Brazil). Glucose and sodium bicarbonate (NaHCO_3_) were obtained from Dinâmica (Brazil). Hydrochloric acid (HCl), potassium monobasic phosphate (KH_2_PO_4_) and sodium hydroxide (NaOH) were purchased from Nuclear (Brazil). Solutions of these substances, except for glucose, NaCl, and NaHCO_3_, were prepared in distilled water and kept under refrigeration.

1,1-Diphenyl-2-picrylhydrazyl (DPPH), apocynin, arachi donic acid (AA), carbamylcholine hydrochloride (carbachol, CCh), dithiothreitol, eosin, ethylenediaminetetraacetic acid (EDTA), formaldehyde, histamine dihydrochloride, iberiotoxin (IbTX), isoproterenol, Mayer’s hematoxylin, methane hydroxymethylamine (Tris), nifedipine, ovalbumin (OVA) (grade V), tempol and Tween 20 were purchased from Sigma-Aldrich (Brazil). Catalase was obtained from Cayman Chemical (Brazil) and saline (0.9% NaCl) was from Fresenius Kabi LTDA (Brazil). The carbogen mixture (95% O_2_ and 5% CO_2_) was acquired from White Martins (Brazil).

All reagents used in sodium dodecyl sulfate-polyacrylamide gel electrophoresis (SDS-PAGE), Bradford’s reagent, Laemmli’s buffer, sodium fluoride; sodium pyrophosphate, sodium vanadate and Triton X 100 were purchased from Bio-Rad (Richmond, CA). Nitrocellulose membranes and kits for chemiluminescence detection were obtained from Amersham (United Kingdom). Anti-ERK1/2, anti-SOD and anti-β-actin antibodies were obtained from Santa Cruz Biotechnology (Santa Cruz, CA, United States), anti-PI3K antibody from Abcam plc. (United Kingdom) and biotinylated goat anti-rabbit secondary antibody from Vector Laboratories (Burlingame, CA, United States).

The reagents used in the immunohistochemical analysis, including ABC Kit by Vectastain PK-6102 (anti-mouse) and the chromogen 3,3′-diaminobenzidine (3,3′-DAB), were purchased from Dako (United States). Harris hematoxylin was obtained from Merck.

### Induction of Chronic Allergic Lung Inflammation in Guinea Pig

The animals were randomly divided into two groups (5–8 animals/group): control (Ctrl) and chronic allergic lung inflammation (Asth). For nebulization, the guinea pigs were individually placed in a closed polyacrylic box coupled to an ultrasonic nebulizer. Soon after, they were nebulized with a saline solution of OVA for a maximum of 15 min or until the onset of respiratory distress, defined as the presence of sneezing, coryza, coughing and/or drawing of the thoracic wall; Ctrl received only saline. The time that the guinea pigs remained in nebulization was defined as inhalation time.

**FIGURE 1:**
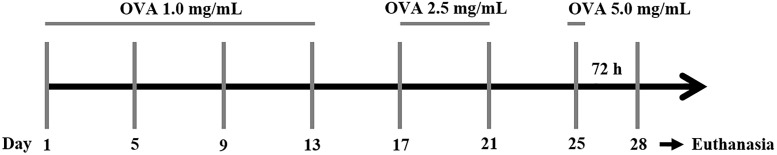
Protocol for induction of chronic allergic lung inflammation in guinea pigs. OVA: ovalbumin.

The protocol consisted of seven inhalations performed over a period of 4 weeks at 96-h intervals between each inhalation, with OVA concentration being increased (1 to 5 mg/mL) to avoid tolerance. In the first four inhalations, the guinea pigs were subjected to 1 mg/mL OVA; in the fifth and sixth inhalations, the animals received OVA 2.5 mg/mL; and in the seventh inhalation, OVA concentration was increased to 5 mg/mL. At 72 h after the last inhalation, the animals were euthanized by cervical dislocation followed by sectioning of cervical vessels for experimentation (Figure 1). The control animals were subjected to the same inhalation procedure but received only saline (adapted from Tibério et al., 1997; Angeli et al., 2008; Pigati et al., 2015).

### Evaluation of Chronic Allergic Lung Inflammation Effects on Lung and Bronchial Morphology of Guinea Pigs

The animals were euthanized by guillotine, and the lung and the right extrapulmonary bronchus were isolated, fixed in 10% formaldehyde for 24 h. The tissues were then subjected to standard histological procedures as follows: (1) dehydration with an increasing alcohol series of 70% for 24 h, 80, 96, and 100% (three baths) for 1 h each; (2) clearing in 100% xylene:alcohol (1:1) for 1 h, followed by two baths in pure xylene for 1 h each; and (3) embedding in paraffin by passing the sample through two baths of liquid paraffin (heated to 50°C) for 1 h each. Then, the samples were embedded in new paraffin. The blocks obtained were cut to 5 μm thick and stained with Mayer’s hematoxylin/eosin (Howard et al., 2004). For a panoramic analysis of the histological section, the slides were analyzed at ×40 (1500 μm) and ×100 (600 μm) magnification.

The aspects evaluated were bronchial wall structure, tissue integrity and cell migration. For each experimental group, 5 slides of 5 different animals were analyzed. The photomicrographs of the slides were captured with a camera coupled to a light microscope. The images were calibrated using the Motic Plus program according to the objectives used: 4× and 10×. The histological analysis of the slides was performed by a trained operator who qualitatively analyzed the histological parameters and then evaluated them statistically by score (below).

1. Preserved histoarchitecture of the airways with the absence of perivascular and peribronchiolar cellular infiltration;
2. Mild degree: an increase of less than 25% compared to control;
3. Moderate degree: an increase of 25 to 49% compared to control;
4. Pronounced degree: an increase of 50 to 75% compared to control; and
5. Very pronounced degree: an increase of more than 75% compared to control.

### Evaluation of Chronic Allergic Lung Inflammation Effects on Guinea Pig Nebulization Time

The time elapsed for guinea pigs to begin showing signs of respiratory distress during inhalation time, which lasted up to a maximum of 15 min, was assessed, as well as the development of respiratory restrictions such as dyspnea or apnea (Tibério et al., 1997). Comparisons were then made between the two groups.

### Preparation of Guinea Pig Trachea

The animals were euthanized as described in Section “Evaluation of Chronic Allergic Lung Inflammation Effects on Lung and Bronchial Morphology of Guinea Pigs.” The trachea was immediately removed and cleaned of fat and connective tissue; the tracheal rings (2–3 cm) were immersed in Krebs solution and bubbled with carbogen mixture (95% O_2_ and 5% CO_2_). The Krebs solution composition was (mM): NaCl (118.0), KCl (4.55), MgSO_4_ (5.7), KH_2_PO_4_ (1.1), CaCl_2_ (2.52), NaHCO_3_ (25.0) and glucose (11.0), with pH adjusted to 7.4. To record isometric contractions, tracheal segments were suspended in steel rods in organ baths (6 mL), connected to a force transducer (TIM 05), attached to an amplifier (AECAD04F) and connected to an A/D converter in a computer running AQCAD^®^ software (São Paulo, Brazil). The system contained a thermostatic pump model BT 60 that controlled the organ bath temperature. The trachea resting time was 60 min in a preload tension of 1 g (baseline). During the organ resting phase, the solution was changed every 15 min to avoid metabolite accumulation (Tschirhart et al., 1987).

After the stabilization period, the tracheal rings were contracted with 1 μM CCh and isometric tension was recorded. When a stable contraction was attained, 0.1 mM arachidonic acid was added to the organ bath to confirm the presence of epithelium by the presence of arachidonic acid-induced relaxation equal to or greater than 50% of maximal tension. In some tracheal rings, the luminal surface was gently rubbed with Krebs-moistened cotton tip to remove the epithelial layer. The absence of epithelium was confirmed when arachidonic acid-induced relaxation was absent or lower than 10% of maximal tension (Tschirhart et al., 1987).

### Contractile Reactivity Measurement

#### Evaluation of Contractile Response to Ovalbumin in Guinea Pig Trachea in the Presence of Functional Epithelium

The trachea was assembled as previously described. After the stabilization period, when baseline remained constant, the intact epithelium tracheal rings were stimulated with 10 μg/mL OVA (Schultz, 1910; Dale, 1913; Chand and Eyre, 1978; Jones et al., 1988), and the contraction amplitude was compared between the two groups. The maximum mean amplitude (measured in gram-force of tension) obtained for the control group was set as 100%, and the Asth group had their E_max_ calculated by comparing their maximum amplitude with that of the control. All the contraction experiments were unpaired.

#### Evaluation of Contractile Response to CCh, Histamine, and KCl in Guinea Pig Trachea in Both the Presence and Absence of Functional Epithelium

The trachea was assembled as described in Section “Preparation of Guinea Pig Trachea.” After the evaluation of epithelium integrity, a cumulative concentration-response curve was obtained to CCh (10 nM to 0.1 mM), a muscarinic receptor agonist (Ouedraogo and Roux, 2014), histamine (10 nM to 3 mM), a histaminergic receptor agonist (Ouedraogo and Roux, 2014) or KCl (1 mM to 0.3 M), a contractile agent of electromechanical mechanism (Du et al., 2006).

In some experiments, the curves to CCh were obtained in the absence and presence of the following: 10 μM apocynin, an inhibitor of NADPH oxidase (Sutcliffe et al., 2012), and 1 mM tempol, a superoxide dismutase mimetic (De Boer et al., 2001) after 30 min incubation; and 100 IU/mL catalase (Rhoden and Barnes, 1989) after 10 min incubation.

Contractile reactivity was evaluated on the basis of the maximum effect (E_max_) and the negative logarithm of the molar concentration of a substance that produced 50% of its maximal effect (pEC_50_) of the contractile agent, calculated from the concentration-response curves obtained. The maximum mean amplitude (measured in gram-force of tension) obtained for the control group was set at 100%, and the other groups had their E_max_ calculated by comparing their maximum amplitude with that of the control. The comparisons were made only between preparations with and without epithelium, not making cross-comparisons (e.g., Ctrl without epithelium vs. Asth with epithelium). For the comparison between the preparations in the absence and presence of pharmacological tools, each group had its own control, referred to as 100%. All the contraction experiments were unpaired. All experiments were performed using the middle third of the trachea, avoiding the initial and final thirds, to prevent the effect of differences in the expression of receptors in the tracheal tissue, as described by Daniel et al. (1986) and Leff (1988).

### Relaxant Reactivity Measurement

The trachea was assembled as described in Section “Preparation of Guinea Pig Trachea.” After the epithelium integrity evaluation, we induced a contraction with 1 μM CCh and performed a cumulative concentration-response curve to isoproterenol (1 pM to 3 μM), a β-adrenergic receptors agonist (Naline et al., 1994), or nifedipine (10 pM to 1 mM), a Ca_V_ blocker (Ahmed et al., 1985).

In some experiments, the curves to isoproterenol were obtained in the absence and presence of 10 μM SB431542, a type 1 TGF-β receptor antagonist (Inman et al., 2002), after 60 min incubation, and 0.1 μM IbTX, a selective BK_Ca_ blocker (Yagi et al., 2002), after 20 min incubation.

Relaxant reactivity was expressed as the reverse percentage of the initial contraction force elicited by CCh and evaluated on the basis of pEC_50_ and E_max_, calculated from the obtained concentration-response curves.

### Evaluation of Antioxidant Activity in Plasma and Lung

#### Plasma and Lung Tissue Preparation

After euthanasia, blood was collected by cardiac puncture. Next, the blood was placed in test tubes containing EDTA to obtain the plasma. In addition, lungs were homogenized with KCl (1:1). The samples were then centrifuged at 1198 ×*g* for 10 min, and the supernatant was transferred to Eppendorf tubes and stored at -20°C (Okafor et al., 2011; Silva et al., 2012).

#### Total Antioxidant Capacity Analysis

The procedure was based on the colorimetric analysis of DPPH reduction (Brand-Williams et al., 1995). Briefly, 1.25 mg DPPH were dissolved in 100 mL of ethanol and the solution kept under refrigeration and protected from light. DPPH solution, 2 mL, and 100 μL of the sample were added to proper centrifuge tubes. The tubes were vortexed and allowed to stand for 30 min. They were then centrifuged at 7489 ×*g* at 20°C for 15 min, and the supernatant was read in a spectrophotometer at 515 nm (Biospectro, model SP-220/Brazil). Results were expressed as the percentage of oxidation inhibition: AOA = 100 – [(DPPH⋅R)_S_/(DPPH⋅R)_W_ × 100]. Where (DPPH⋅R)_S_ and (DPPH⋅R)_W_ correspond to the concentration of DPPH⋅ remaining after 30 min, measured in the sample (S) and blank (B) prepared with distilled water.

### Western Blot Analysis

Lung samples were collected, homogenized in a protein extraction buffer (100 mM Tris, 10% sodium dodecyl sulfate, 100 mM sodium pyrophosphate, 100 mM sodium fluoride, 10 mM EDTA and 10 mM sodium orthovanadate) and boiled for 5 min. The tissue extracts were then centrifuged at 17,530 ×*g* at 4°C for 40 min. Protein determination of the supernatants was performed by the Bradford method (Bio-Rad Laboratories, Hercules, CA, United States). The proteins were treated with *Laemmli* sample buffer containing 200 mM dithiotreitol. The aliquots (50 μg) were subjected to 6.5% SDS-10% PAGE. Electro-transfer of proteins from the gel to a nitrocellulose membrane was performed for 2 h at 120 V in a Bio-Rad miniature transfer apparatus (Towbin et al., 1992).

The membranes were blocked for 2 h at room temperature with blocking solution (5% skim milk). The nitrocellulose membranes were incubated overnight at 4°C with primary antibodies anti-p85 PI3K (catalog number #4228, Cell Signaling, MA, United States), anti-ERK 1/2 (catalog number sc-292838, Santa Cruz, Dallas, TX, United States) or anti-p-ERK 1/2 (catalog number sc-81492, Santa Cruz, Dallas, TX, United States) diluted in blocking buffer with 3% albumin added, and then washed for 30 min in buffer without milk. The blots were subsequently incubated with peroxidase-conjugated secondary antibody for 1 h. Immunoblot visualization was carried out with the enhanced chemiluminescence system (Amersham Biosciences). The immunoblots were scanned and quantified using ImageJ software (imagej.net/Downloads).

### Imunohistochemical for α-Actin Analysis

The lungs were obtained and paraffin-embedded, as described in Section “ Evaluation of Chronic Allergic Lung Inflammation Effects on Lung and Bronchial Morphology of Guinea Pigs.” The tissue sections were deparaffinized, rehydrated and washed three times for 10 min each with 3% H_2_O_2_ 10 V to inhibit endogenous peroxidase activity. Actin was detected with anti-α-actin antibody (dilution: 1:600; Santa Cruz Biotechnology, CA, United States – cod. SC-22613) for the detection of human smooth muscle actin markers (Dako). Sections of experimental and control (positive and negative) slides were incubated overnight with primary antibody. The following day, antigen retrieval for the smooth muscle actin marker was performed at high temperature in a citrate solution at pH 6.0 for 30 min. After this treatment, the sections, still immersed in the same solution, were cooled to room temperature for 20 min, washed in PBS three times and incubated with a secondary antibody using the ABC Kit by Vectastain (PK-6102, anti-mouse). The chromogen used was 3,3′-DAB (Dako). Finally, the sections were washed in water and counterstained with Harris hematoxylin (Merck). Optical density was used to evaluate the volume fractions of α-actin. Images were captured using a Leica DM2500 microscope (Leica Microsystems, Wetzlar, Germany), a digital camera (Leica DFC420 Leica Microsystems) and the image analysis software Image-Pro Plus 4.5 (Media Cybernetics, Bethesda, MD, United States). This software allowed a threshold of color shades to be developed. These shades represented the positive areas quantified in the previously determined area. Ten fields at ×400 magnification were evaluated per animal, and positive areas in the airway were expressed as a percentage of the total airway area (Camargo et al., 2018).

### Statistical Analysis

Results were expressed as the mean and standard error of the mean (SEM) and statistically analyzed using Student’s *t-*test for single comparison or one-way analysis of variance (ANOVA) followed by the Tukey’s post-test for multiple comparisons. Values were significantly different when *p* < 0.05. All data were analyzed by GraphPad Prism^®^ version 5.01 (GraphPad Software Inc., San Diego, CA, United States).

## Results

### Evaluation of Chronic Allergic Lung Inflammation Effects on Lung and Bronchial Morphology of Guinea Pigs

**FIGURE 2:**
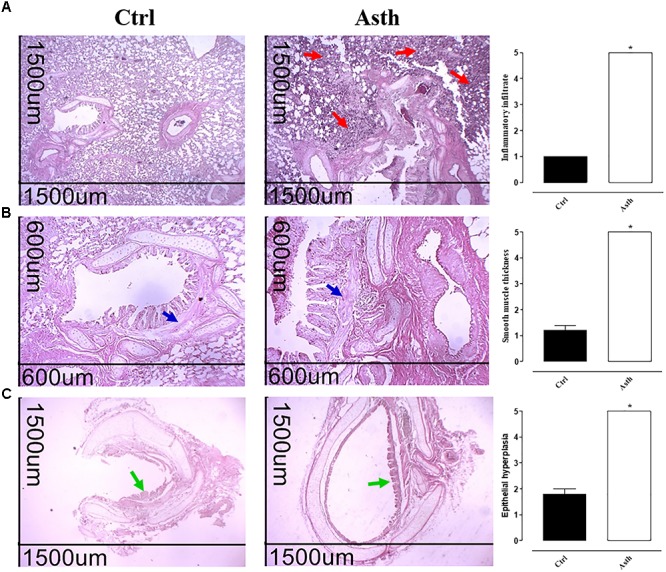
Photomicrographs of lung from guinea pigs in Ctrl and Asth groups, demonstrating infiltration of inflammatory cells in the peribronchial region and score of peribronchial cellular infiltrate **(A)**, bronchial smooth muscle and score of muscle layer thickness **(B)**, and bronchial epithelium and score of epithelial hypertrophy **(C)**. Cellular infiltrate (red arrows), intrapulmonary bronchial smooth muscle (blue arrows), and bronchial epithelium (green arrows). Hematoxylin/eosin, ×40 (1500 μm) and ×100 (600 μm). The columns and vertical bars represent the mean and SEM, respectively (*n* = 5). Student’s *t-*test, ^∗^*p* < 0.05 (Ctrl vs. Asth). Ctrl: control group; Asth: chronic allergic lung inflammation group.

The histological analysis allowed an evaluation of the morphological structure of blood vessels, intra-and extrapulmonary bronchi, bronchioles and pulmonary alveoli (Figure 2, *n* = 5).

At ×40 magnification, the histological sections of the lungs obtained from Ctrl showed a normal histological appearance and preserved pulmonary histoarchitecture. Meanwhile, the lungs from Asth (*p* < 0.05) showed large amounts of peribronchiolar and perivascular inflammatory cell infiltration and absence of infiltrates in the pulmonary alveoli (Figure 2A, *n* = 5). In addition, images at ×100 confirmed with greater structural detail the alterations seen at ×40. In this case, epithelial hyperplasia and intrapulmonary bronchial smooth muscle thickness were increased in animals in Asth (*p* < 0.05), compared to Ctrl (Figure 2B, *n* = 5).

The histological sections of the extrapulmonary bronchi at ×40 revealed that animals in Asth displayed bronchial smooth muscle development due to hypertrophy and/or hyperplasia (*p* < 0.05), compared to the histological sections of Ctrl (Figure 2C, *n* = 5).

### Evaluation of Chronic Allergic Lung Inflammation Effects on Guinea Pig Nebulization Time

The inhalation period of 900 s was standardized for guinea pigs in Ctrl (Table 1, *n* = 8). In the first four nebulizations, the animals in Asth had similar nebulization times as Ctrl. However, chronic allergic lung inflammation reduced the times of the fifth (456.4 ± 96.7 s), sixth (400.8 ± 51.0 s) and seventh (273.3 ± 26.6 s) nebulizations in relation to Ctrl (900 s) (*p* < 0.05) (Table 1, *n* = 8).

**Table 1:** Mean time of contact (s) of guinea pigs with ovalbumin from the first to fourth (1–4), fifth (5), sixth (6) and seventh (7) nebulizations.

Group	1–4	5	6	7
Ctrl	900.0	900.0	900.0	900.0
Asth	900.0	456.4 ± 96.7^∗#^	400.8 ± 51.0^∗#^	273.3 ± 26.6^∗#^

*One-way ANOVA followed by Tukey’s post-test: ^∗^p < 0.05 (1–4 vs. 5/6/7 intragroups); ^#^p < 0.05 (Ctrl vs. Asth). Ctrl: control group; Asth: chronic allergic lung inflammation group (n = 8).*

No signs of respiratory distress were observed in guinea pigs in Ctrl. In contrast, animals in Asth exhibited coughing, sneezing, dyspnea and thoracic drainage, especially after the seventh nebulization.

### Contractile Reactivity Measurement

#### Evaluation of the Contractile Response to Ovalbumin in Guinea Pig Trachea in the Presence of Functional Epithelium

**FIGURE 3:**
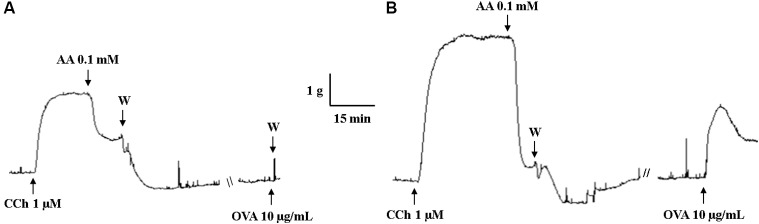
Representative records of guinea pig trachea response to stimulation with 10 μg/mL OVA in Ctrl **(A)** and Asth **(B)**. CCh: carbachol; AA: arachidonic acid; W: washout; OVA: ovalbumin; Ctrl: control group; Asth: chronic allergic lung inflammation group.

The guinea pig trachea of Ctrl had no contractile response to OVA stimulation (E_max_ = 1.8 ± 0.8%) (Figure 3). Differently, chronic allergic lung inflammation caused a significant contractile response (E_max_ = 100%) (*p* < 0.05) (Figure 3).

#### Evaluation of Contractile Response to CCh, Histamine, and KCl in Guinea Pig Trachea in the Presence and Absence of Functional Epithelium

**FIGURE 4:**
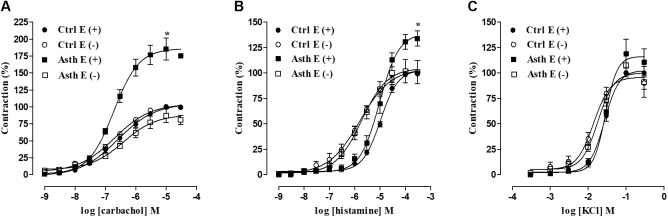
Cumulative concentration-response curves to CCh **(A)**, histamine **(B)** and KCl **(C)** in guinea pig trachea in Ctrl and Asth, both in the presence and absence of functional epithelium. The symbols and vertical bars represent the mean and SEM, respectively (*n* = 5). ANOVA one-way followed by Tukey’s post-test: ^∗^*p* < 0.05 [Ctrl E (+) vs. Asth E (+)]. Ctrl: control group; Asth: chronic allergic lung inflammation group; E (+): epithelium present; E (–): epithelium absent.

In Ctrl, guinea pig trachea contracted in response to the addition of cumulative concentrations of CCh (1 nM to 0.1 mM) in both the presence (E_max_ = 100%; pEC_50_ = 6.63 ± 0.10) and absence of functional epithelium (E_max_ = 100%; pEC_50_ = 6.85 ± 0.09). Chronic allergic lung inflammation increased the contractile efficacy of CCh (E_max_ = 185.3 ± 16.1%) (*p* < 0.05), but it did not alter its potency (pEC_50_ = 6.74 ± 0.03) in the presence of functional epithelium; meanwhile, in the absence of epithelium there was no difference in either the efficacy (E_max_ = 89.2 ± 9.8%) or potency of CCh (pEC_50_ = 6.62 ± 0.07), compared to Ctrl (Figure 4A, *n* = 5).

Moreover, guinea pig trachea from Ctrl contracted in response to the addition of cumulative concentrations of histamine (10 nM to 3 mM) in both the presence (E_max_ = 100%; pEC_50_ = 5.05 ± 0.04) and absence of functional epithelium (E_max_ = 100%; pEC_50_ = 5.80 ± 0.15). Chronic allergic lung inflammation also increased histamine contractile efficacy in the presence of epithelium (E_max_ = 132.6 ± 5.9%) (*p* < 0.05) but did not alter its potency (pEC_50_ = 5.11 ± 0.12). In the absence of epithelium, there was no difference in contractile efficacy (E_max_ = 102.2 ± 11.4%) or potency (pEC_50_ = 5.80 ± 0.15) of histamine between the two groups.

Intragroup comparison of the agonists’ effects showed that, in Ctrl and Asth, there was a difference in histamine contractile potency between the tracheal rings with and without epithelium; in this case, the agonist was more potent in the absence of epithelium, about 6.4-fold in Ctrl and 4.2-fold in Asth (*p* < 0.05) (Figure 4B, *n* = 5).

Furthermore, guinea pig trachea in Ctrl contracted in response to the addition of cumulative concentrations of KCl (1 mM to 0.3 M) in both the presence (E_max_ = 100%; pEC_50_ = 1.60 ± 0.01) and absence of functional epithelium (E_max_ = 100%; pEC_50_ = 1.89 ± 0.08). Chronic allergic lung inflammation did not alter the contractile effectiveness or potency of KCl, either in the presence (E_max_ = 119.0 ± 14.2%; pEC_50_ = 1.56 ± 0.03) or absence of functional epithelium (E_max_ = 107,6 ± 11.5%; pEC_50_ = 1.73 ± 0.07). However, in Ctrl, the potency of KCl was 1.8-fold higher in the absence of epithelium (*p* < 0.05), not differing in Asth (Figure 4C, *n* = 5).

#### Evaluation of ROS Participation in the Alterations Induced by Chronic Allergic Lung Inflammation

**FIGURE 5:**
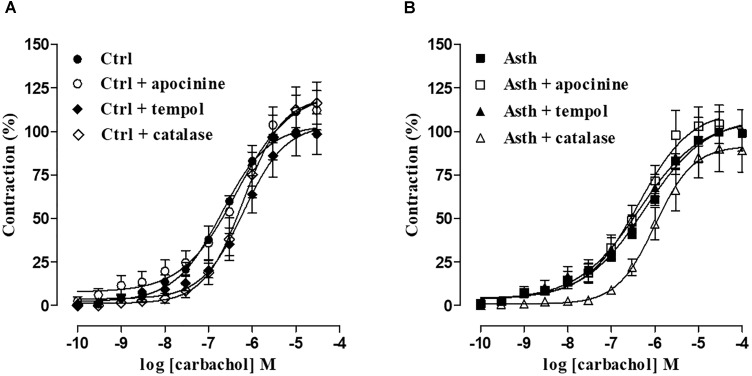
Cumulative concentration-response curves to CCh in guinea pig trachea with epithelium in Ctrl **(A)** and Asth **(B)**, in both the absence and presence of apocynin, tempol or catalase. The symbols and vertical bars represent the mean and SEM, respectively (*n* = 5). ANOVA one-way followed by Tukey’s post-test: ^∗^*p* < 0.05 [Ctrl E (+) vs. Asth E (+)]. Ctrl: control group; Asth: chronic allergic lung inflammation group; E (+): epithelium present.

In Ctrl, the cumulative concentration-response curve to CCh (0.1 nM to 30 μM) (E_max_ = 100%; pEC_50_ = 6.82 ± 0.06) was not altered in the presence of apocynin, either in relation to efficacy (E_max_ = 115.6 ± 9.4%) or potency (pEC_50_ = 6.52 ± 0.13), in guinea pig trachea in the presence of epithelium. In contrast, in the presence of tempol, the contraction curve to CCh (0.1 nM to 30 μM) was 3.2-fold shifted to the right (pEC_50_ = 6.30 ± 0.07) (*p* < 0.05), with no alteration in the contractile effectiveness of the agonist (E_max_ = 99.4 ± 12.3%). Similarly, in the presence of catalase, the contractile potency of CCh (0.1 nM to 0.1 mM) was reduced 4-fold (pEC_50_ = 6.23 ± 0.09) (*p* < 0.05), with no effect on its efficacy (E_max_ = 116.6 ± 11, 9%) (Figure 5A, *n* = 5).

In guinea pigs with chronic allergic lung inflammation (Asth), there were no changes in either efficacy or potency of CCh (0.1 nM to 0.1 mM) (E_max_ = 100%; pEC_50_ = 6.35 ± 0.11) induced by apocynin (E_max_ = 105.6 ± 11.7%; pEC_50_ = 6.54 ± 0.11) in guinea pig trachea in the presence of epithelium. Similarly, in the presence of tempol, the contraction curve to CCh (0.1 nM to 0.1 mM) was not modified (E_max_ = 101.0 ± 11.7%; pEC_50_ = 6.62 ± 0.27), but the potency of CCh (0.1 nM to 0.1 mM) was reduced about 3-fold in the presence of catalase (E_max_ = 102.4 ± 9.1%; pEC_50_ = 5.99 ± 0.06) (*p* < 0.05) (Figure 5B, *n* = 5).

### Relaxant Reactivity Measurement

#### Evaluation of the Relaxant Response to Isoproterenol and Nifedipine in Guinea Pig Trachea in the Presence and Absence of Functional Epithelium

**FIGURE 6:**
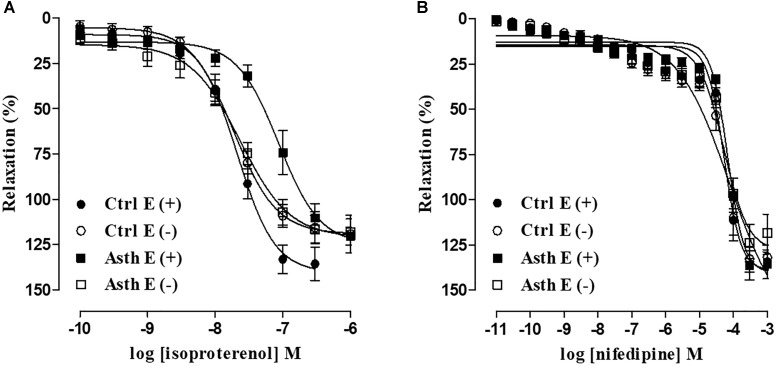
Cumulative concentration-response curves to isoproterenol **(A)** and nifedipine **(B)** in guinea pig trachea pre-contracted with 1 μM CCh in Ctrl and Asth, in both the presence and absence of functional epithelium. The symbols and vertical bars represent the mean and SEM, respectively (*n* = 5). Ctrl: control group; Asth: chronic allergic lung inflammation group; E (+): epithelium present; E(–): epithelium absent.

In Ctrl, isoproterenol (0.1 nM to 1 μM) relaxed the guinea pig trachea pre-contracted with 1 μM CCh in both the presence (E_max_ = 135.7 ± 9.1%; pEC_50_ = 7.79 ± 0.10) and absence of functional epithelium (E_max_ = 115.9 ± 8.7%; pEC_50_ = 7.81 ± 0.09). Chronic allergic lung inflammation did not alter the relaxant efficacy of isoproterenol in either the presence (E_max_ = 124.4 ± 6.6%) or absence of functional epithelium (E_max_ = 118.2 ± 7.3%). However, agonist relaxant potency was reduced about 3.5-fold in Asth (pEC_50_ = 7.24 ± 0.09) (*p* < 0.05) compared to Ctrl in the presence but not absence of epithelium (pEC_50_ = 7.89 ± 0.12) (Figure 6A, *n* = 5).

In addition, nifedipine (1 pM to 1 mM) relaxed the guinea pig trachea in Ctrl pre-contracted with 1 μM CCh in both the presence (E_max_ = 138.2 ± 8.3%; pEC_50_ = 4.54 ± 0.15) and absence of functional epithelium (E_max_ = 132.9 ± 7.2; pEC_50_ = 4.73 ± 0.10). Chronic allergic lung inflammation did not alter the efficacy or relaxant potency of nifedipine, either in the presence (E_max_ = 122.8 ± 8.8%; pEC_50_ = 4.54 ± 0.15) or absence of functional epithelium (E_max_ = 123.9 ± 10.4%; pEC_50_ = 4.71 ± 0.23). There was no difference in the efficacy or relaxant potency of nifedipine between the preparations with and without epithelium in either Ctrl or Asth (Figure 6B, *n* = 5).

#### Evaluation of the Participation of Transforming Growth Factor β (TGF-β) and Big Conductance Ca^2+^-Sensitive K^+^ Channels (BK_Ca_) in the Alterations Induced by Chronic Allergic Lung Inflammation

**FIGURE 7:**
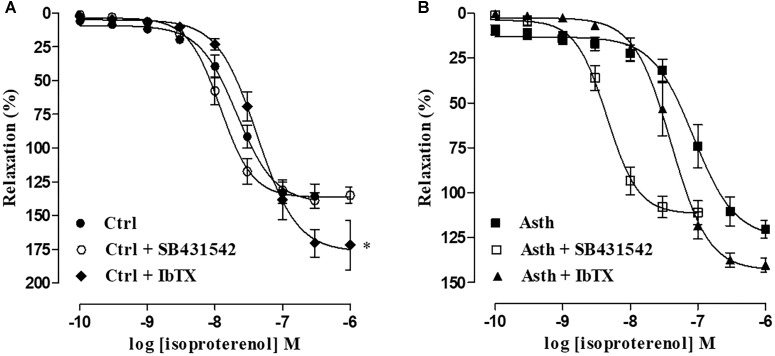
Cumulative concentration-response curves to isoproterenol in guinea pig trachea with epithelium pre-contracted with 1 μM CCh in Ctrl **(A)** and Asth **(B)**, in both the absence and presence of SB431542 or IbTX. The symbols and vertical bars represent the mean and SEM, respectively (*n* = 5). Ctrl: control group; Asth: chronic allergic lung inflammation group; E (+): epithelium present. IbTX: iberiotoxin.

In Ctrl, the relaxation curve of isoproterenol (0.1 nM to 1 μM) (E_max_ = 135.7 ± 9.1%; pEC_50_ = 7.79 ± 0.10) was not altered by SB431542 (E_max_ = 132.4 ± 7.8%; pEC_50_ = 7.96 ± 0.08), but its potency was reduced (pEC_50_ = 7.39 ± 0.06) and its efficacy increased (E_max_ = 175.7 ± 11.0%) by IbTX (*p* < 0.05) (Figure 7A, *n* = 5). However, in animals with chronic allergic lung inflammation, the relaxation curve of isoproterenol (0.1 nM to 1 μM) (E_max_ = 120.7 ± 5.2%; pEC_50_ = 7.24 ± 0.09) was potentiated about 7-fold by SB431542 (pEC_50_ = 8.38 ± 0.08) (*p* < 0.05), with no change in its relaxant efficacy (E_max_ = 108.9 ± 5.6%); and furthermore, both potency and efficacy were unaltered by IbTX (E_max_ = 137.5 ± 3.9%; pEC_50_ = 7.44 ± 0.12) (Figure 7B, *n* = 5).

### Evaluation of Antioxidant Activity in Plasma and Lung

**FIGURE 8:**
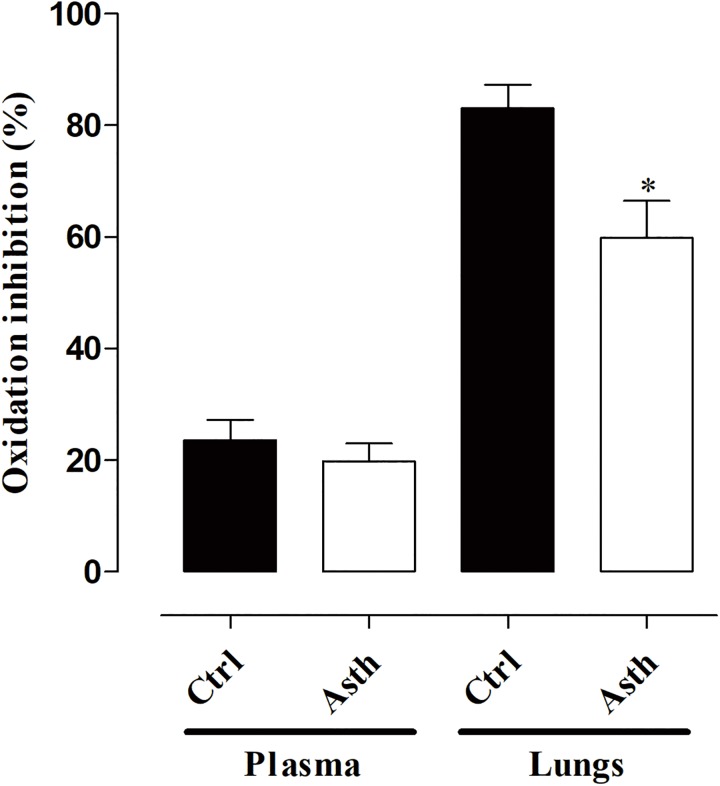
Measurement of oxidation inhibition in plasma and lung in Ctrl and Asth. The columns and vertical bars represent the mean and SEM, respectively (*n* = 5). Ctrl: control group; Asth: chronic allergic lung inflammation group.

Total antioxidant capacity in plasma was similar in Ctrl (23.6 ± 3.6%) and Asth (19.8 ± 3.2%). Meanwhile, in the lung, it was decreased from 83.0 ± 4.2% (Ctrl) to 59.8 ± 6.6% in Asth (*p* < 0.05) (Figure 8, *n* = 5).

### Western Blot Analysis

**FIGURE 9:**
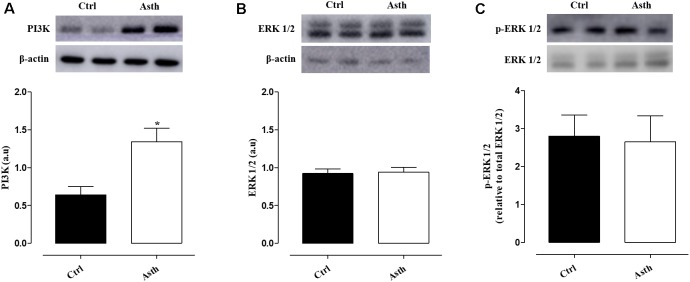
Expression of PI3K **(A)**, ERK 1/2 **(B)**, and p-ERK 1/2 **(C)** proteins in the lung of guinea pigs in Ctrl and Asth. The columns and vertical bars represent the mean and SEM, respectively (*n* = 5). a.u.: arbitrary units. Ctrl: control group; Asth: chronic allergic lung inflammation group; PI3K: phosphatidylinositol-3-kinase; ERK 1/2: extracellular regulated kinase 1/2; p-ERK 1/2: phosphorylated ERK 1/2.

Compared to Ctrl, PI3K expression levels (0.64 ± 0.11 a.u.) were increased in Asth (1.34 ± 0.18 a.u.) (*p* < 0.05). Conversely, ERK 1/2 (0.92 ± 0.06 a.u) and p-ERK (2.80 ± 0.56 a.u.) levels were not altered in the animals by lung inflammation (0.93 ± 0.07 and 2.67 ± 0.68 a.u., respectively) (Figure 9, Supplementary Figure 1, *n* = 4).

### Immunohistochemical for α-Actin Analysis

**FIGURE 10:**
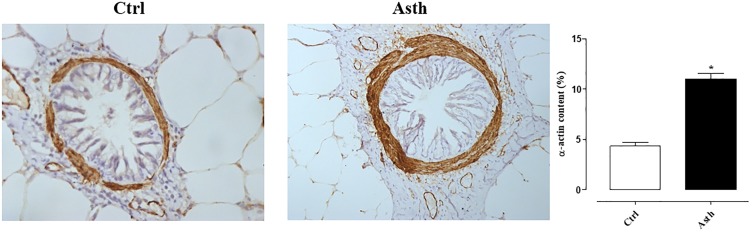
Representative photomicrographs of guinea pig airways immunostained with anti-α-actin antibody in Ctrl **(A)** and Asth **(B)** groups, and its content (%). The columns and vertical bars represent the mean and SEM, respectively (*n* = 5). ×40 (1500 μm). Ctrl: control group; Asth: chronic allergic lung inflammation group.

There were differences in smooth muscle-specific α-actin present in the airway walls between groups. We found increased α-actin content in the airways of the lung inflammation group (11.0 ± 0.53%) compared to the control group (4.32 ± 0.38%) (*p* < 0.05) (Figure 10).

## Discussion

In this work, a model of chronic allergic lung inflammation based on repeated and prolonged exposure of the guinea pig respiratory tract to OVA was standardized, with generation of smooth muscle hypercontractility in response to antigenic challenge, muscarinic and histaminergic receptor stimulation, and relaxant hyporesponsiveness to β-adrenergic agonist, dependent on epithelium-induced release of mediators, ROS generation, reduction in pulmonary antioxidant capacity associated with a decrease in SOD levels, as well as increased PI3K and ERK 1/2 expression and smooth muscle α-actin content. In this sense, the major contribution of this work is the characterization of a model, already used in scientific studies, that reproduces the characteristics of remodeling, smooth muscle hyperreactivity and thickening of the airways. In addition, since this model produces increased airway resistance (Nakashima et al., 2008; Possa et al., 2012), these findings may be associated with *in vivo* bronchial hyperresponsiveness data already described in previous works.

The acute and chronic phases of asthma have different functional and tissue characteristics, that is structural remodeling, due to extensive and repeated animal exposure to inhaled antigenic agents, the marker of the disease chronicity (Westerhof et al., 2001). This pathophysiological event is more related to clinical asthma (Tibério et al., 1997). In this sense, a model of chronic allergic lung inflammation was reproduced, on the basis of OVA inhalation in guinea pigs at increasing concentrations for 4 weeks to obtain an experimental model that mimics the increased contractile reactivity associated with lower airway smooth muscle relaxant responsiveness, providing a valuable tool in studies of asthma pathophysiology and in new pharmacological approaches as well.

By morphological analysis, a peribronchial infiltrate of inflammatory cells was observed, besides some characteristics of tissue remodeling, namely epithelial cell hyperplasia and thickening of the bronchial smooth muscle layer, probably due to the hyperplasia and/or hypertrophy process (Figures 2A–C). These findings agree with similar occurrences found in allergic asthma in humans (Cohen et al., 2007; Bergeron et al., 2009). Also, since persistent inflammation, subsequent remodeling and bronchial hyperesponsiveness have a strong cause and effect relationship and are cardinal characteristics in asthma, these findings are of clinical relevance (Montuschi and Barnes, 2011).

Type I hypersensitivity mediates many of the asthmatic reactions, including airway hyperresponsiveness triggered by antigenic stimulation. This mechanism is related to the capture of immunoglobulins E (IgE) by mast cells, dendritic cells and eosinophils, which release the contractile agents histamine, cys-LTs (LTC_4_, D_4_, and E_4_), contractile prostanoids prostaglandin D_2_ and F_2α_ (PGD_2_ and PGF_2α_, respectively) and thromboxane A_2_ (TXA_2_), thus causing bronchial obstruction (Fireman, 2003). Therefore, the allergenic stimulation of sensitized animals by OVA inhalation is an effective method for evaluating airway hyperresponsiveness.

Animals with lung inflammation had a shorter time in contact with the antigen in the fifth, sixth and seventh inhalations, since this time was sufficient to trigger the respiratory stress signals resulting from the acute response after antigenic exposure, consistent with a profile of bronchial hyperresponsiveness (Table 1), which is corroborated by previous data (Tibério et al., 1997; Pigati et al., 2015). This bronchial hyperresponsiveness may occur due to increased smooth muscle mass, the release of mediators from autonomic nerve endings or innate immune cells. This confirms the last hypothesis that guinea pig trachea showed contractile response to *in vitro* OVA stimulation (Figure 3) on the basis of the Schulz-Dale reaction, in which smooth muscle in an antigenically sensitized animal contracts after exposure to antigen by releasing contractile mediators from microenvironmental cells (Schultz, 1910; Dale, 1913; Shaukat et al., 2015).

The primary function of airway smooth muscle cells is to regulate the tone and narrowing of the airways, controlling intrapulmonary ventilation and consequently the ventilation perfusion ratio (Mitchell, 2009; Ouedraogo and Roux, 2014). The ability to modify smooth muscle length in experiments with isolated organs is useful in demonstrating important features of airway smooth muscle contractile function. In this sense, contradictory results are observed when studies evaluating the contractile response of smooth muscle to different constrictor agents in different animal models of asthma are summarized. Hypercontractility was observed for histamine (Bai, 1991), cholinergic stimuli (Matusovsky et al., 2016) and KCl (Chesné et al., 2015). However, hypocontractility has also been observed for histamine (Whicker et al., 1988) and cholinergic stimuli (Goldie et al., 1986). This variability can be explained by the phenotypic differences of asthma, the experimental model adopted, and the tissue preparation as well (Wright et al., 2013).

In view of the smooth muscle role in bronchial hyperresponsiveness, we investigated guinea pig trachea reactivity in response to contractile stimuli. In guinea pigs with lung inflammation compared to non-sensitized animals, there was an increase in tracheal reactivity to CCh and histamine but not to KCl, only in the presence of functional epithelium (Figures 4A–C). This indicates a greater participation of pharmacomechanical coupling in hypercontractility of the guinea pig trachea, without alteration in the electromechanical component, where this effect depends on epithelial functionality. Moreover, contractile reactivity increased more for CCh than histamine results from cholinergic stimulation being more pronounced in the central airways, such as the trachea (Leff et al., 1988).

It has been demonstrated that the concomitant reduction of airway hyperresponsiveness and smooth muscle mass are correlated, suggesting that such structural changes could explain the functional change in smooth muscle contractile responsiveness (Danek et al., 2004). Thus, these data corroborate the previous morphological ones.

It was observed that the greatest amplitude of contraction to CCh occurred only in the preparations with intact epithelium. It is well described in the literature that epithelial damage resulting from allergic inflammation results in epithelial mediator production, such as TGF-β (Davies, 2009), which mediates smooth muscle hypertrophy in asthma and promotes faster and more sustained contraction (Boxall et al., 2006). These cells are also a source of contractile factors that contribute to bronchoconstriction, such as LTB_4_, LTC_4_, LTD_4_, LTE_4_, and PGD_2_ (Montuschi, 2008; Santini et al., 2016). Since the animals were subjected to chronic stimulation with OVA, which leads to tissue structural and functional changes, it is reasonable to say that the increase in the contractile response was related to the epithelium effect because of its release of hypertrophic mediators.

Additionally, airway smooth muscle function is modulated by ROS. H_2_O_2_ and increased molecular oxygen levels induce contraction of guinea pig trachea (Rhoden and Barnes, 1989) and promote augmented reactivity to contractile agonists, such as ACh and serotonin (Henricks and Nijkamp, 2001). It has also been reported that 8-isoprostane, a marker of oxidative stress, formed by lipid peroxidation, contributes to the greater activity of the airway smooth muscle. In addition, this marker has been found in high concentrations in exhaled breath condensate of asthmatic patients (Montuschi et al., 2010).

Therefore, we have shown that apocynin, an inhibitor of NADPH oxidase (Sutcliffe et al., 2012), did not alter the cumulative curve of CCh in guinea pig trachea preparations in the Ctrl and Asth groups, indicating that O_2_^-^ from NADPH oxidase does not play an important role in the modulation of the contractile tone of guinea pig trachea, regardless of whether there is a profile of allergic lung inflammation. In contrast, the SOD mimetic tempol (De Boer et al., 2001) reduced the contractile potency of CCh in animals of the Ctrl but not Asth group. This suggests that ROS, especially O_2_^-^, is increased because of lung inflammation, since tempol was unable to prevent its action in the tracheal smooth muscle, but that this increase would occur through pathways independent of NADPH oxidase, such as xanthine oxidase and decoupled endothelial nitric oxide (eNOS), which are also responsible for the formation of the O_2_^-^. Moreover, although catalase, an enzyme that converts H_2_O_2_ to water and molecular oxygen (Rhoden and Barnes, 1989), reduced the contractile potency of CCh about 4-fold in Ctrl, the efficacy of catalase in reducing the potency of CCh was lower in guinea pigs with lung inflammation, indicating an increase in H_2_O_2_ production triggered by the inflammatory process (Figures 5A,B).

Indeed, Mátyás et al. (2002) showed that superoxide anion, hydroxyl radicals, and hydrogen peroxide cause the tracheal smooth muscle to contract, the latter being the ROS responsible for the greatest increase in muscle tone. In addition, the effects of these free radicals are contained by antioxidant defenses present in the intact epithelium, and epithelial damage potentiates the contractile effect of these ROS. Therefore, it is reasonable to infer that an imbalance between ROS and antioxidant defenses favors airway smooth muscle damage. However, the role of oxidative stress was not directly investigated by measuring biomarkers of oxidative stress (e.g., H_2_O_2_ or nitrates reflecting peroxynitrite production), so this is a limitation of the study.

Airway hyperresponsiveness is also related to a lower release of smooth muscle relaxants, such as adrenaline (Barnes, 1986), vasoactive intestinal peptide (Ben-Jebria et al., 1993) and prostaglandin E_2_ and I_2_ (PGE_2_ and PGI_2_, respectively) (Van Schoor et al., 2000). Desensitization of β_2_ adrenergic receptors is also associated with increased smooth muscle responsiveness to contractile stimuli in asthma (Barnes, 1995).

The relaxant airway smooth muscle responsiveness evaluation in animal models of asthma has shown hyporresponsiveness to theophylline and β_2_ agonists (Nesmith et al., 2014). In agreement with these data, we observed a reduction in the relaxant potency of isoproterenol in guinea pig trachea in the Asth group, compared to Ctrl, in an epithelium-dependent manner (Figure 6A). In contrast, there were no differences in either efficacy or potency between Ctrl and Asth in nifedipine relaxant response (Figure 6B), indicating that lung inflammation did not promote alterations in the activity of Ca_V_, which was confirmed by the previous KCl cumulative contraction data.

M_3_ receptors crucially influence the function of these receptors by activation of protein kinase C (PKC) and direct phosphorylation of β_2_ receptors, as well as G_rms_ protein, promoting receptor decoupling with its G protein, as well as desensitization (Meurs et al., 2001); in addition to phosphorylation by a G-protein receptor kinase (GRK), the β-adrenoceptor kinase (BARK), amplifying the desensitization of these receptors (Boterman et al., 2006). Additionally, ACh acting via M_2_ receptors counteracts the bronchodilator actions of β_2_ agonists by reducing the cAMP levels due to inhibition of adenylate cyclase (AC) (Montuschi and Ciabattoni, 2015). Such processes explain the well-known attenuation of the efficacy of β-agonists during episodes of severe bronchoconstriction (Meurs et al., 2008). Furthermore, TGF-β, a key growth factor involved in some remodeling features, and released by cholinergic stimulation (Oenema et al., 2013) has been shown to reduce the number of β-adrenoceptors in human tracheal smooth muscle cells (Nogami et al., 1994), so this process is closely related to the lower relaxant responsiveness due to the inactivation of the G_s_/BK_Ca_ pathway, either by β-adrenergic desensitization, by TGF-β or M_3_-PKC, or by negative modulation of these channels via the pathway triggered by cholinergic stimulation of M_2_-G_iβγ_/BK_Ca_ (Kume, 2015).

We then showed that the relaxation curve of isoproterenol in control animals was not modified by the antagonist of type I TGF-β receptors as expected, since the antagonist has no effect in the absence of agonist. However, the potency of the agonist was decreased by the BK_Ca_ blocker, as expected since in non-asthmatic airway smooth muscle, relaxation via β-receptors is also mediated via the opening of BK_Ca_ (Kume, 2013), but also had its maximum response increased, probably due to a compensatory mechanism. Furthermore, in the trachea of the Asth group, the antagonist recovered the relaxant potency of isoproterenol, indicating that signaling mediated via TGF-β RI leads to desensitization of β-receptors (Figures 7A,B). These data are confirmed by previous research which showed that lung inflammation produces a greater release of TGF-β in the lungs (Possa et al., 2012). Additionally, the BK_Ca_ blocker did not modify the relaxation curve, indicating that this inability is due to the fact that these channels are already poorly activated because of lung inflammation, contributing to the observed less responsiveness of the relaxant.

In summary, we found that as lung inflammation increased the contractility of guinea pig trachea, it also reduced the relaxant reactivity of this organ by mechanisms related to ROS generation and the release of TGF-β. Confirming the influence of ROS, we showed that allergic inflammation caused a decrease in the antioxidant capacity of lungs, but not in the plasma, indicating local pulmonary damage rather than systemic damage (Figure 8).

As demonstrated, lung inflammation promoted thickening of the smooth muscle layer, which may have occurred due to hypertrophy and/or hyperplasia. These cell growth processes are dependent on two independent signaling pathways that regulate the growth of airway smooth muscle cells, phosphatidylinositol-3-kinase (PI3K) and extracellular regulated kinase 1/2 (ERK 1/2) (Gosens et al., 2008). These pathways can be switched on by the activation of G protein-coupled receptors or tyrosine kinase receptors by contractile mediators or growth factors, such as the TGF-β (Hoshino et al., 2001). Thus, we showed that pulmonary expression of PI3K was increased in the Asth group, but not total or phosphorylated ERK 1/2, confirming the participation of this kinase in pulmonary inflammation, which could be associated with hypertrophy/hyperplasia induced by allergic lung inflammation (Figure 9).

It is well established that airway inflammation and cholinergic stimulation promote the release of TGF-β, which induces airway remodeling (Oenema et al., 2013), which, in turn, induces increased expression of smooth muscle contractile proteins such as α-actin, smooth muscle myosin (SM-myosin) and myosin light chain kinase (MLCK; Fairbank et al., 2008). Accordingly, α-actin content has been shown to be increased almost 3-fold by pulmonary inflammation (Figure 10), indicating that thickening of the smooth muscle layer is related to observed contractile hyperresponsiveness. Given the mechanisms investigated, we can assume that this increase is due to the participation of the epithelium, which acts by releasing ROS and TGF-β.

Unlike the current models for the study of asthma in animals, the model provided here is characterized by smooth muscle hypercontractility due to ROS and growth factor release, as well as activation of PI3K. Furthermore, this work will promote advances in asthma research on bronchial hyperresponsiveness and will also contribute to the search for new therapeutic strategies in this field.

## Author Contributions

LV and MS performed pharmacological experiments, analyzed data, completed the literature review and designed the manuscript. AC and GO engaged in the pharmacological experiments. IS participated in pharmacological experiments and prepared the manuscript. FQ and PdS involved in histological evaluation and data acquisition. LA and CC participated in the acquisition of protein expression data. GC and AS performed the antioxidant capacity experiments, and analyzed and interpreted the data. RR and IT carried out the immunohistochemical assays. GV performed the histological analysis. FC and BS involved in the design of the study, interpretation of data and paper review.

## Conflict of Interest Statement

The authors declare that the research was conducted in the absence of any commercial or financial relationships that could be construed as a potential conflict of interest.
