# Supplementary material for: A Guinea Pig Model of Airway Smooth Muscle Hyperreactivity Induced by Chronic Allergic Lung Inflammation: Contribution of Epithelium and Oxidative Stress
Source: Front Pharmacol. 2019 Jan 24;9:1547. doi: 10.3389/fphar.2018.01547 (PMC6353839; doi:10.3389/fphar.2018.01547)
Supplement: Supplementary file 1 [file Data_Sheet_1.pdf]

Supplementary Material

A guinea pig model of airway smooth muscle hyperreactivity induced by chronic allergic lung inflammation: contribution of epithelium and oxidative stress

Luiz Henrique César Vasconcelos, Maria da Conceição Correia Silva, Alana Cristina Costa, Giuliana Amanda de Oliveira, Iara Leão Luna de Souza, Fernando Ramos Queiroga, Layanne da Cunha Araujo, Glêbia Alexa Cardoso, Renato Fraga Righetti, Alexandre Sérgio Silva, Patrícia Mirella da Silva, Carla Roberta de Oliveira Carvalho, Giciane Carvalho Vieira, Iolanda de Fátima Lopes Calvo Tibério, Fabiana de Andrade Cavalcante, Bagnólia Araújo da Silva

***Correspondence:** Bagnólia Araújo da Silva, Laboratório de Farmacologia Funcional Prof. George Thomas, Programa de Pós-graduação em Produtos Naturais e Sintéticos Bioativos, Centro de Ciências da Saúde, Universidade Federal da Paraíba, Cidade Universitária, João Pessoa/PB, Zip Code 58.051-970, P.O. Box 5009, Brazil. bagnolia@ltf.ufpb.br

1 Supplementary Figures and Tables

1.1 Supplementary Figures

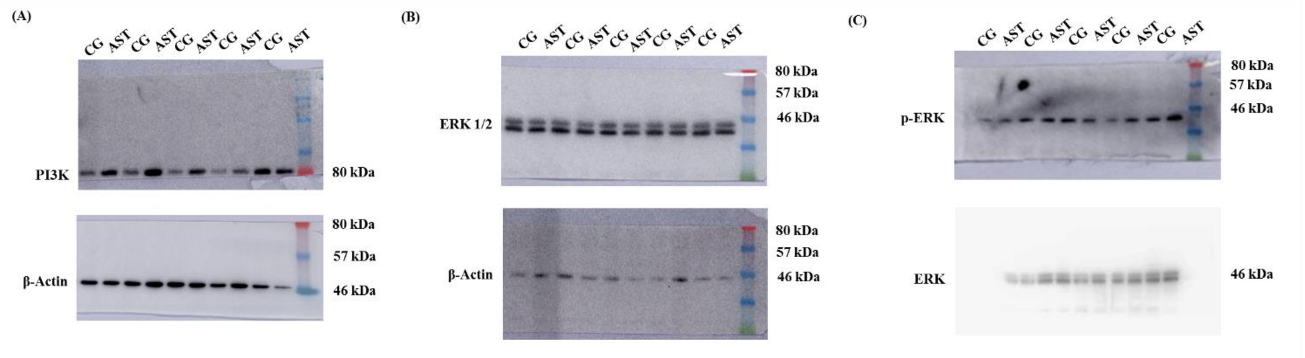

Supplementary Figure 1. Original records of Western blot for expression analysis of PI3K (A), ERK 1/2 (B) and p-ERK 1/2 (C) proteins in the lung of guinea pigs in Ctrl and Asth. Ctrl: control group; Asth: chronic allergic lung inflammation group; PI3K: phosphatidylinositol-3-kinase; ERK 1/2: extracellular regulated kinase 1/2; p-ERK 1/2: phosphorylated ERK 1/2.
